# The Impact of the Renin-Angiotensin-Aldosterone System on Inflammation, Coagulation, and Atherothrombotic Complications, and to Aggravated COVID-19

**DOI:** 10.3389/fphar.2021.640185

**Published:** 2021-06-17

**Authors:** M. Ekholm, T. Kahan

**Affiliations:** Karolinska Institutet, Department of Clinical Sciences, Danderyd Hospital, Division of Cardiovascular Medicine, Stockholm, Sweden

**Keywords:** hypertension, haemostasis, coagulation, renin-angiotensin-aldosterone system, angiotensin II, antihypertensive treatment

## Abstract

Atherosclerosis is considered a disease caused by a chronic inflammation, associated with endothelial dysfunction, and several mediators of inflammation are up-regulated in subjects with atherosclerotic disease. Healthy, intact endothelium exhibits an antithrombotic, protective surface between the vascular lumen and vascular smooth muscle cells in the vessel wall. Oxidative stress is an imbalance between anti- and prooxidants, with a subsequent increase of reactive oxygen species, leading to tissue damage. The renin-angiotensin-aldosterone system is of vital importance in the pathobiology of vascular disease. Convincing data indicate that angiotensin II accelerates hypertension and augments the production of reactive oxygen species. This leads to the generation of a proinflammatory phenotype in human endothelial and vascular smooth muscle cells by the up-regulation of adhesion molecules, chemokines and cytokines. In addition, angiotensin II also seems to increase thrombin generation, possibly *via* a direct impact on tissue factor. However, the mechanism of cross-talk between inflammation and haemostasis can also contribute to prothrombotic states in inflammatory environments. Thus, blocking of the renin-angiotensin-aldosterone system might be an approach to reduce both inflammatory and thrombotic complications in high-risk patients. During COVID-19, the renin-angiotensin-aldosterone system may be activated. The levels of angiotensin II could contribute to the ongoing inflammation, which might result in a cytokine storm, a complication that significantly impairs prognosis. At the outbreak of COVID-19 concerns were raised about the use of angiotensin converting enzyme inhibitors and angiotensin receptor blocker drugs in patients with COVID-19 and hypertension or other cardiovascular comorbidities. However, the present evidence is in favor of continuing to use of these drugs. Based on experimental evidence, blocking the renin-angiotensin-aldosterone system might even exert a potentially protective influence in the setting of COVID-19.

## Introduction

Cardiovascular disease is the most common cause of death in economically developed countries, and is expected to remain a leading cause globally due to an increasing prevalence in developing countries (Mathers and Loncar, 2006). Given the high morbidity and mortality burden in cardiovascular disease, it is important for societies and their health care systems to improve strategies to decrease the incidence of cardiovascular disease (Leal et al., 2006). Traditional risk factors cardiovascular disease include hypertension, hypercholesterolemia, diabetes mellitus, obesity, tobacco smoking, age, male sex, and family history. These risk factors contribute to endothelial dysfunction, and inflammation plays a key role in early-stage endothelial dysfunction and oxidative stress in the vascular wall, which are hallmarks of subclinical atherosclerosis (Rafieian-Kopaei et al., 2014; Castellon and Bogdanova, 2016).

The renin-angiotensin-aldosterone system (RAAS) is of vital importance in the pathobiology of vascular disease. Angiotensin (Ang) II promotes atherosclerosis (Nickenig, 2002) and has important impact on vascular inflammation and haemostasis. Ang II has been shown to cause oxidative stress, induce endothelial dysfunction and to generate a proinflammatory phenotype in human vascular smooth muscle cell (VSMC)s by stimulating up-regulation of the adhesion molecules, chemokines and cytokines (Schieffer et al., 2000; Brasier et al., 2002). Ang II is also pivotal in vascular remodeling, as it induces the expression of a number of growth factors (Satoh et al., 2001). In addition, Ang II modulates vascular cell migration and growth (Yaghini et al., 2010), decreases VSMC apoptosis (Li et al., 2006), alters extracellular matrix modulation composition (Wesselman and De Mey, 2002), and Ang II has been shown to initiate and accelerate hypertension, endothelial dysfunction and atherosclerosis (Weiss et al., 2001). Conversely, inhibition of the RAAS components decreases experimental atherosclerosis (Rader and Daugherty, 2008; Lu et al., 2012), and death from cardiovascular disease in humans (Yusuf et al., 2000). Thus, it has been proposed that RAAS inhibition may have anti-atherosclerotic effects beyond the effects of the blood pressure reduction (Yusuf et al., 2000).

Ang II also has an impact on haemostasis and may increase thrombin generation. Inflammatory stimuli can prime the coagulation system through several mechanisms, and the cross-talk between inflammation and haemostasis helps to explain prothrombotic states in inflammatory environments (Verhamme and Hoylaerts, 2009). The coagulation cascade in inflammatory states is mainly mediated by tissue factor (TF) (Cimmino et al., 2011). Under physiological conditions TF is not expressed or is in an inactive state in circulatory or endothelial cells (Rao et al., 2012). However, various inflammatory signals, like cytokines, can induce TF activation in endothelial cells (Szotowski et al., 2005). Ang II may increase TF expression in vessels, also platelet activation by circulating Ang II may have an important contributing role in the generation of thrombin (Burger et al., 2011; Cordazzo et al., 2013).

**FIGURE 1:**
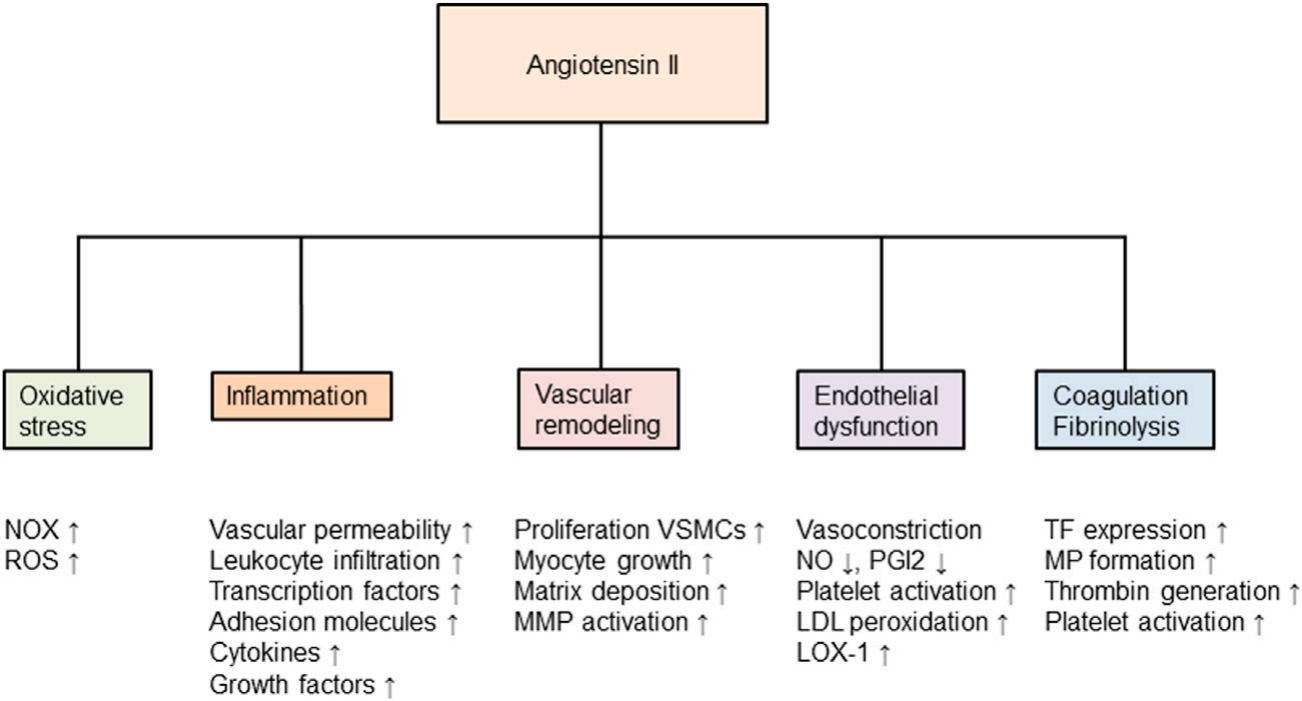
Angiotensin II and its effects in the development of atherosclerosis. Nox, nicotinamide-adenine dinucleotide phosphate oxidase; ROS, reactive oxygen species; MMP, matrix metalloproteinase; VSMCs, vascular smooth muscle cells; NO, nitric oxide; PGI2, prostaglandin I_2_ (also called prostacyclin); LDL, low-density lipoprotein; LOX, lectin-like oxidized low-density lipoprotein receptor; TF, tissue factor; MP, microparticles and tPA, tissue plasminogen activator. Modified from (Volpe, 2012).

There are multiple effects of Ang II on the atherosclerotic process, as summarized in Figure 1. This review, however, will focus on the RAAS and events associated with inflammation, oxidative stress, endothelial dysfunction. Second, we discuss the influence of the RAAS on the haemostatic system via cross-talk between inflammation and the haemostatic system, and through a direct effect of Ang II on thrombosis. Finally, the relationship to the corona virus disease 2019 (COVID-19) to the RAAS, and the known complications of this infection, are discussed.

## The Endothelium

### The Importance of Healthy and Intact Endothelium

The endothelium has an important function in preserving a physiological structure and function. Endothelial cells form a monolayer that produces factors that regulate vascular tone, inflammation, haemostasis, vascular cell growth and death, angiogenesis, the migration of leukocytes, and prevents platelet adhesion (Gawaz et al., 2005). Vascular tone is dependent on a delicate balance between vascular dilators, such as NO, prostaglandin I_2_ (also called prostacyclin); and endothelium derived hyperpolarizing factor and vascular constrictors, such as Ang II, endothelin-1, and thromboxane A_2_ (Sandoo et al., 2010). Also, VSMCs are affected by endothelial cells and other factors, and VSMCs can themselves release cytokines and growth-regulatory mediators, which in turn have an impact on vessel phenotype and growth.

In inflammatory conditions, endothelial cells release procoagulant and antifibrinolytic mediators, like von Willebrand factor, thromboxane A_2_ and plasminogen activator inhibitor-1 (PAI-1). Activated endothelial cells express TF and adhesion molecules, which are of vital importance in mediating the interaction of leukocytes and platelets with endothelial cells, and thus activating the coagulant system and promoting an inflammatory response. The cytokines interleukin (IL)-1, IL-6, IL-8, and tumor necrosis factor α (TNF-α), and also the chemokine monocyte chemoattractant protein-1 (MCP-1), and the growth factor transforming growth factor-β1 (TGF-β1), have all been shown to play a key role in mediating the procoagulant changes in endothelial dysfunction (van der Poll et al., 1994; Ceciliani et al., 2002).

## Inflammation and Oxidative Stress

### The Inflammatory Process

Several mediators of inflammation are up-regulated in subjects with atherosclerotic disease (Kalsch et al., 2007). For diagnostic use, the cytokine IL-6, and C-reactive protein (CRP), have generated considerable attention. CRP is generated by hepatic cells and is modulated by IL-6, but also by TNF-α and IL-1β (Calabró et al., 2003), thereby contributing to the up-regulation of MCP-1 and selectins, such as P- and E-selectin and the cell adhesion molecules intracellular adhesion molecule -1 (ICAM-1) and vascular cell adhesion molecule-1 (VCAM-1). CRP attenuates the synthesis of endothelial NO (Verma et al., 2002), and causes augmented PAI-1 (Devaraj et al., 2003). Increased concentrations of acute phase reactants like CRP, IL-6, leukocyte count and fibrinogen are all associated with an increased risk of cardiovascular disease (Ridker et al., 1998; Ridker et al., 2000; Kaptoge et al., 2012). Also, phospholipase A2, which is implicated in the oxidation of low-density lipoprotein (LDL) and subsequent oxidative stress and inflammation, can predict atherosclerotic disease (Di Angelantonio et al., 2012).

The infiltration of inflammatory cells is associated with the secretion of mediators like TGF-β1 and highly pro-inflammatory interleukins like IL-1β and IL-18 (Dinarello, 2018). This stimulates excessive extracellular matrix fibroblasts to differentiate into active myofibroblasts (Wynn, 2008). A key mediator to this process is TGF-β1, and its release can be promoted by Ang II (Wynn, 2008; Meng et al., 2016). The major sources of TGF-β1 are macrophages, monocytes, neutrophils, myofibroblasts, and epithelial cells (Wynn and Ramalingam, 2012), and during states with prolonged activation of Ang II and TGF-β1 a chronic inflammation contributes to adverse effects and diseases like chronic heart failure (Gullestad et al., 2012). In addition, Ang II, TGF-β1, and IL-1β also supress activity of matrix metalloproteinases, which contributes to scar tissue and stiffening (Porter and Turner, 2009).

Inflammasomes have an important role in cardiovascular disease, in particular the subtype nucleotide-binding oligomerization domain, leucine-rich repeat and pyrin domains-containing protein 3 (NLRP3) (Pinar et al., 2020). This inflammasome complex consists of three intracellular subunits, the NLRP3 sensor protein, the apoptosis-associated speck-like protein (ASC), and pro-caspase-1. Upon activation NLRP3 first recruits ASC and then pro-caspase-1. This in turn leads to pro-caspase-1 being cleaved to active caspase-1, which cleaves pro- IL-1β and pro-IL-18 to mature inflammatory cytokines (Man and Kanneganti, 2015). Active caspase-1 in turn cleaves gasdermin-D to an N-terminal fragment that can signal transduce promoting inflammation. NLRP3 can be activated by a wide range of danger signals, such as mitochondrial dysfunction, extracellular adenosine triphosphate and damaged nucleic acids, but also by Ang II and TGF-β1 trigger a number of pattern recognition receptors (Suthahar et al., 2017).

### The Cell Reduction-Oxidation Reaction State

Oxidative stress is an imbalance between anti- and prooxidants, with a subsequent increase of ROS, leading to tissue damage. Thus, the cell redox state is an important factor of endothelial cell biology. Traditional risk factors for cardiovascular disease can initiate endothelial dysfunction by changing the cell redox state and, consequently, the oxidative stress in the vascular wall. Increased generation of superoxide anion, O2ˉ and, subsequently, oxidative stress results in enhanced catabolism of NO, endothelial dysfunction and impaired vasodilatation. Reactive oxygen species (ROS) also has the ability to reduce the activity of NO synthase (NOS) and to increase the breakdown of NO. Also, NO is a potent endogenous inhibitor of VSMC migration and growth (Sarkar et al., 1996) and, in higher concentrations, impairs up-regulation of adhesion molecules and cytokines (Qian and Fulton, 2012).

The transcription of the pleiotropic nuclear factor kappa-light-chain-enhancer of activated B-cells (NF-ҡB) has a pivotal role in endothelial up-regulation of cytokines and adhesion molecules. In the nucleus NF-ҡB binds to target genes coding for proinflammatory and proatherogenic proteins, and promotes gene transcription (Moynagh, 2005). NO is a powerful inhibitor of activation of NF-ҡB (Grumbach et al., 2005). It is to be noted that NO and the superoxide anion reacts to form the powerful oxidant peroxynitrite, ONOOˉ, that can damage endothelial cells. Reactive oxygen species also lowers the availability of tetrahydrobiopterin. If this occurs, the oxygenase function of NOS is replaced by its reductase function and ROS are produced instead of NO, which increases NF-ҡB activity, and the expression of cytokines (Mittal et al., 2014). Thus, the ratio between ROS and NO regulates the redox state and is of vital importance for proper function of the vascular endothelium. An imbalance between NO and ROS increases the risk for vasospasm, VSMC proliferation, and can cause an imbalance between tissue plasminogen activator (tPA) and PAI-1, which may predispose a prothrombotic state.

## Tissue Factor

### Coagulation Cascade

The TF pathway (or the extrinsic pathway) and the contact pathway (or the intrinsic pathway), divides the coagulation cascade into two parts. The TF pathway is the major inducer of the coagulation cascade of the two under healthy conditions. The TF pathway is initiated when TF is bound to FVII-FVIIa. The TF-FVIIa complex cleaves FX into FXa, and FIX into FIXa, respectively. FXa then forms the prothrombinase complex when associated with FVa and Ca^2+^ on activated platelets, resulting in thrombin formation (Grover and Mackman, 2018). The reaction is much faster in the presence of negatively charged surface phospholipids (i.e., phoshatidylserine) on activated platelet membranes. Microparticles may also accelerate thrombin generation due to a procoagulant phosphatidylserine-rich surface that supports thrombin generation (Davizon and Lopez, 2009).

### Tissue Factor Circulates in Three Pools

TF is present in the extravascular space (subendothelial tissue) and not exposed to flowing blood, except during plaque rupture. However, TF is also present in circulating blood, mainly in three pools. The most important source of TF is monocytes, expressed as membrane-anchored and membrane-bound. There is no evidence that granulocytes express TF; rather they seem to acquire TF from monocytes (Egorina et al., 2008). Controversial is the expression of TF in platelets. Human platelets appear not to express TF when activated (Osterud and Olsen, 2013). Platelets might store TF in *α*-granules or acquire TF from TF-containing microparticles (Bouchard et al., 2010; Camera et al., 2010). On the other hand, activated platelets generate microparticles that seem to express TF on their surface and contain negatively charged phospholipids, two very important factors for coagulation reactions (Mallat et al., 2000). The second source of circulating TF is different cell-derived microparticles, which are considered to be key players in atherothrombosis. The third pool, probably of minor importance, is a spliced form of TF, which is soluble and circulates in the blood (Bogdanov et al., 2003).

Blood-borne TF means that the coagulation cascade may be activated without contact between the blood and the extravascular space (i.e., plaque rupture). This appears to be an important contribution in intravascular thrombosis (Balasubramanian et al., 2002). Blood-borne TF implicates that TF apparent on the cell surface under normal conditions is in an inactive, cryptic, state. To be functionally active and to exhibit a procoagulant activity, TF has to undergo an activation step, decryption. The importance of the blood-borne TF is still controversial, and the molecular differences between these states remain to be clarified (Rao et al., 2012).

### Inflammation Activates and Decrypts Tissue Factor

The coagulation cascade in inflammatory states is mainly mediated by TF (Cimmino et al., 2011). Various inflammatory signals, like IL-6, IL-1, or TNF-α, can induce TF activation in monocytes and macrophages and endothelial cells. TF expression is mostly dependent of IL-6, and inhibition of IL-6 can completely block thrombin generation (van der Poll et al., 1994). IL-6, but also IL-1β and IL-8 has been shown to be relevant for activation of coagulation (Bester and Pretorius, 2016). Also, CRP seems to have a role in coagulation as CRP can induce TF activation *via* a NF-κB pathway in both endothelial cells and VSMCs (Cirillo et al., 2005). In addition, Other mediators like oxidized LDL (Cimmino et al., 2019) and oxygen free radicals (Cimmino et al., 2015) can enhance TF activation. This enables active TF to be exposed to blood and bind to FVII. The complex of TF and FVIIa then contributes to the conversion of FIX and FX into the active proteases FXa and FXa, thereby generating thrombin. TF also possesses a function as a signaling receptor, where TF binding of FVIIa triggers VSMC proliferation (Cirillo et al., 2004).

## Cross-Talk Between Inflammation and Coagulation

### The Effects of Inflammation on Coagulation

Evidence suggests that inflammation and haemostasis are tightly interrelated in a bidirectional way, i.e., inflammation may lead to activation of the haemostatic system, while the haemostatic system has an impact on the inflammatory activity (Verhamme and Hoylaerts, 2009). This helps to explain prothrombotic states in inflammatory environments. Several trials show the importance of the cytokines such as IL-6, IL-1β, IL-8, TNF-α, and MCP-1 in the activation of coagulation by inflammation, (van der Poll et al., 2001; Levi et al., 2012).

### The Effects of Coagulation on Inflammation

Coagulation factors can induce vascular inflammation by their binding to protease activated receptor (PAR)s, which are present in endothelial cells, leukocytes, platelets, fibroblasts, and VSMCs (Coughlin, 2000). An exceptional characteristic of PARs is that these receptors carry their own ligand, which is unmasked until the receptor is cleaved. PARs are G protein-coupled receptors, and up to date, four receptors have been identified (PAR 1–4). Thrombin is the most essential player in activation of PARs, and can activate PAR-1, PAR-3 as well as PAR-4. Factor (F)Xa transmits activation of PAR-1 to PAR-3, while TF-FVIIa transmits activation of PAR-2 (Coughlin, 2000). PAR activation up-regulates inflammatory molecules, like cytokines, chemokines, growth factors and adhesion molecules. Experiments in healthy human subjects have shown a 4-fold increase in plasma concentrations of IL-8 and IL-6 by recombinant FVIIa (de Jonge et al., 2003). Activation of PARs transforms endothelial cells into a proinflammatory phenotype, causing vascular permeability and local accumulation of platelets and leukocytes (Alberelli and De Candia, 2014). In VSMCs, PAR activation mediates contraction, proliferation, migration, hypertrophy and modulation of the extracellular matrix, thereby contributing to atherosclerosis and hypertension.

## Thrombin, Platelets and Protease-Activated Receptors

### The Impact of Thrombin

**FIGURE 2:**
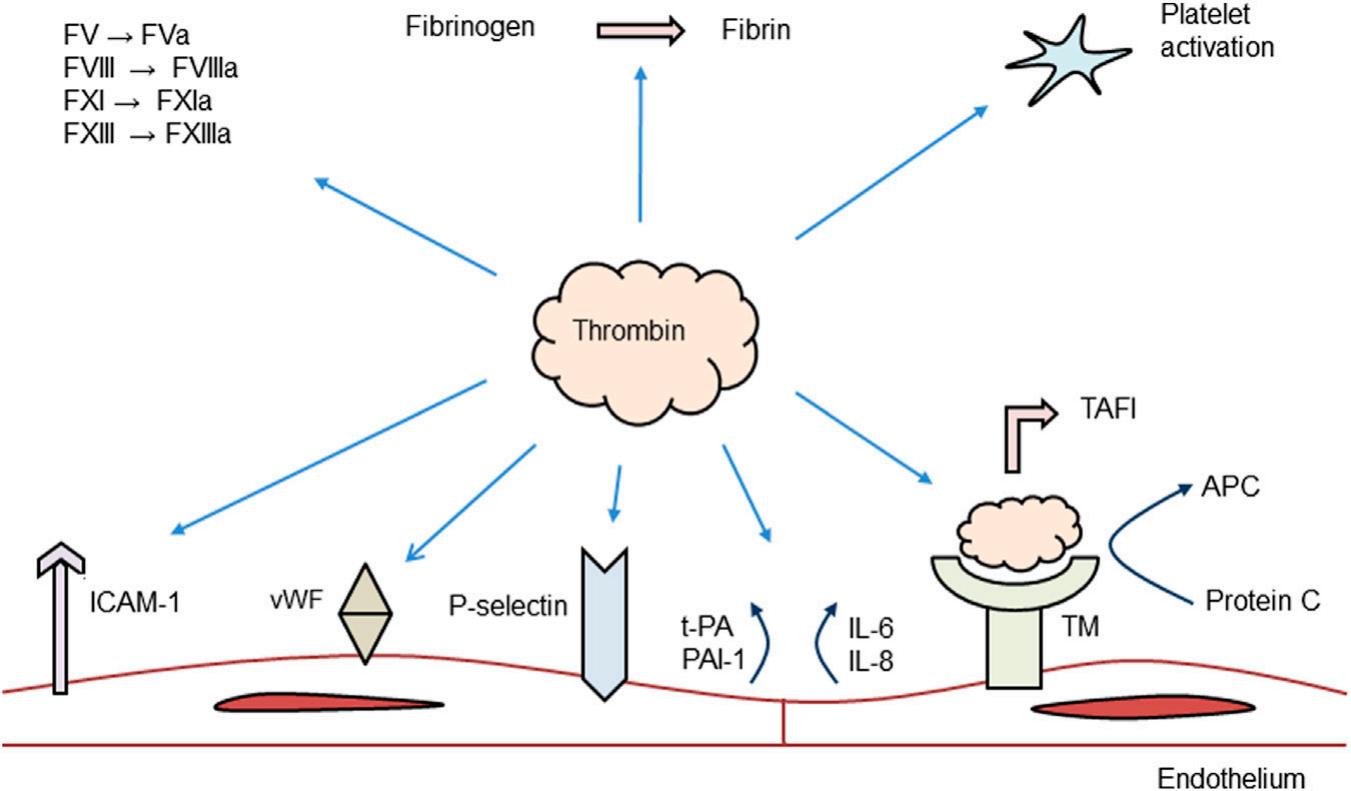
Role of thrombin in the coagulation system. ICAM, intracellular cell adhesion molecule; PAI, plasminogen activator inhibitor; vWF, von Willebrand factor; IL, interleukin; TAFI, thrombin fibrinolysis activatable inhibitor; TM, thrombomodulin; APC, activated protein C; and F, coagulation factor. Modified from (Davidson, 2013).

Figure 2 summarizes the role of thrombin in the coagulation system. The main action of thrombin is to convert fibrinogen to fibrin monomers, the end result of the coagulation cascade. However, thrombin also exercises an anticoagulant role by binding to thrombomodulin at the intact endothelium and to promote activation of the protein C pathway. Activated protein C then inactivates FVa and FVIIIa, two essential cofactors for FXa and FIXa, thereby down-regulating thrombin generation (Esmon, 2003). Thrombin also has a central role in the inflammatory response and stimulates different cell types in the vasculature and in blood, including endothelial cells, VSMCs, and platelets. The effects of thrombin are mediated by its counter-receptors, PARs.

### The Impact of Platelets

Human platelets express PAR-1 and PAR-4. PAR-1 seems to be the main platelet receptor for thrombin (Duvernay et al., 2017). Thrombin is the most effective platelet activator and causes platelets to change shape, secrete a number of substances, mobilize P-selectin and cluster of differentiation 40 ligand (CD40L), leading to activation of αIIbβ3 and, ultimately, aggregations of platelets (Brass, 2003). Thus, thrombin signaling in platelets contributes significantly to haemostasis and thrombosis (Duvernay et al., 2017).

IL-1β is a pivotal mediator in the cytokine cascade and also an important activator of endothelial cells, by inducing the cytokines IL-6, IL-8, and chemokine MCP-1 (Gawaz et al., 2002). Platelet IL-1β also induces up-regulation of adhesion molecules like ICAM-1 and the vitronectin receptor (αvβ3), contributing to adhesion of neutrophils and monocytes to endothelial cells (Gawaz et al., 2000). CD40L is also a key player in platelet activation. CD40L and its receptor CD40 are expressed in cells including platelets, endothelial cells, VSMCs, T lymphocytes and macrophages (Daub et al., 2020). Activated, platelets rapidly (within seconds) express CD40L, and the interaction of CD40L and CD40 on endothelial cells up-regulate the cytokines IL-1, IL-8 and IL-6, MCP-1 and adhesion molecules, as well as increasing TF expression (Antoniades et al., 2009). When platelets and endothelial cells are activated, P-selectin is translocated to the cell surface where it functions as a platelet-selectin glycoprotein ligand-1 receptor, expressed on leukocytes (and in small amounts on platelets). The subsequent P-selectin and platelet-selectin glycoprotein ligand-1 interaction in turn increases the release of cytokines and chemokines from neutrophils and monocytes. The interaction also stimulates the up-regulation of adhesion molecules and TF on endothelial cells and leukocytes (Polgar et al., 2005). Thrombin also promotes the release of endothelial cell microparticles, which seems to be critical in vascular pathophysiology (Sapet et al., 2006). Once trace amounts of thrombin have been generated, this can activate FV, FVIII, FXI, and FXIII.

### The Shift of the Endothelium

In the healthy endothelium, thrombin activates PAR-1 and stimulates the production of NO and prostaglandin I_2_, leading to vasodilatation and fibrinolysis by the release of tPA (Gudmundsdottir et al., 2006). During inflammation and endothelial dysfunction PAR expression is increased, priming the response of endothelial cells to thrombin, and shifting endothelial cells to a proinflammatory phenotype, inducing synthesis and release of PAI-1, contributing to impaired fibrinolysis (Hirano, 2007). In pathophysiological conditions, PAR-1 activation causes morphological changes, an increased vascular leakage, release of proinflammatory cytokines, and up-regulation of adhesion molecules (Rahman et al., 1999). In particular the synthesis of IL-6 seems to mediate the transition of the inflammatory process in the vessels from an acute to a chronic phase (Marin et al., 2001). Activation of PAR-1 also mobilizes P-selectin and von Willebrand factor from Weibel-Palade bodies, promoting endothelial cell rolling and subsequently firm adhesion of both platelets and leukocytes.

In healthy arteries PARs are preferably expressed in endothelial cells, while their expression in VSMCs is limited. In hypertension and in atherosclerotic vessels PAR-1 is up-regulated in VSMCs (Hirano, 2007). This implies that PARs on VSMCs have a more prominent role under pathological conditions. In conditions associated with endothelial dysfunction, PARs in VSMCs mediate contraction, proliferation, migration, hypertrophy and the production of extracellular matrix modulation (Hirano, 2007).

Altogether, in healthy conditions thrombin and PAR-1 activation causes endothelium dependent vasodilatation. In contrast, under pathological conditions, thrombin induces a procoagulatory and proinflammatory state.

## The RAAS and Vascular Complications

### The Components of the RAAS

**FIGURE 3:**
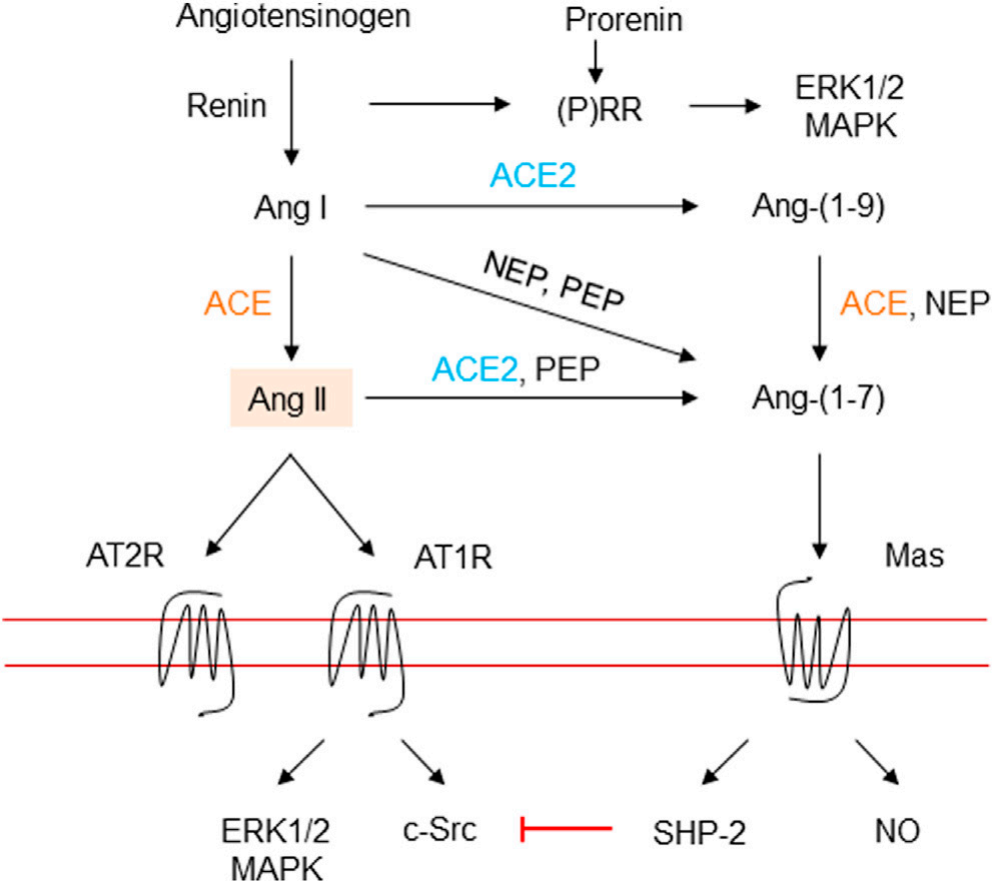
The renin-angiotensin-aldosterone system. Renin is secreted due to various stimuli, and then cleaves angiotensinogen into the inactive decapeptide Ang I. Renin and prorenin can also interact with (pro) renin receptors to activate the MAP kinases ERK1/2 and p38 pathways. ACE cleaves Ang I into the octapeptide Ang II, or to Ang-(1–7) by ACE2 and probably ACE. ACE2 may also produce Ang-(1–7) from Ang II. ACE also inactivates bradykinin into inactive fragments. Importantly, Ang II may be generated directly from angiotensinogen through non-ACE pathways. Ang II activates AT1R, a G protein-coupled receptor. Vasoconstriction and stimulation of aldosterone tend to elevate blood pressure. Ang II also activates AT2R, a G protein-coupled receptor, which can antagonize the effects of activation of the AT1R. Ang, angiotensin; ACE, angiotensin converting enzyme; MAPK, mitogen-activated protein kinase; extracellular signal regulated kinase, ERK; angiotensin II type 1 receptor, AT1R; angiotensin II type 2 receptor, AT2R; (P)RR, (pro)renin receptor; c-Src, cellular Src kinase, a non-receptor tyrosine kinase; SHP-2, Src-homology 2 domain-containing phosphatase 2; NO, nitric oxide; NEP, neutral endopeptidase, and PEP, prolyl endopeptidase.

All RAAS peptides are derived from angiotensinogen, which is synthesized in an abundance in the liver, but angiotensinogen messenger ribonucleic acid (mRNA) has also been detected in many other tissues (Dickson and Sigmund, 2006; Kumar et al., 2012). Figure 3 summarizes the different RAAS components. In the classical pathway, the rate limiting enzyme renin is produced in the kidney (juxtaglomerular cells), and is released in response to vasodilation, low sodium, and by beta_1_-adrenoceptor stimulation. In the blood stream, renin cleaves angiotensinogen into Ang I, which is processed by the membrane bound exopeptidase angiotensin converting enzyme (ACE), to produce Ang II, the predominant peptide of the RAAS (Putnam et al., 2012; Nehme et al., 2019).

The components of the RAAS have dual roles, as the circulating hormone can also be present in several tissues to act as a potential local system (Dell'Italia, 2011; Nehme et al., 2019). A local or tissue RAAS, Ang II production from independent regulation of angiotensinogen in tissue compartments and locally synthesized enzymes may form a local RAAS operating independently of the circulatory RAAS (De Mello and Frohlich, 2011; Sparks et al., 2014). Ang II can then be produced inside cells and consequently the hormone can bind to its receptors in adjacent cells, thus acting in an autocrine/paracrine manner (Kumar et al., 2012). Thus, the local RAAS in distinct tissues acts *via* autocrine and paracrine mechanisms to exacerbate the effects of circulating RAAS and/or works independently to induce a response within tissues.

Local Ang II formation is also dependent on other enzymes such as chymase, tonin, and D and G cathepsin. Chymase, which appears the most important, belongs to the serine proteases and is stored in secretory granules in mast cells. Under normal conditions mature mast cells do not circulate in the bloodstream. They are present throughout the body, and are found in mucosal and epithelial tissues and in all vascularized tissues (Krystel-Whittemore et al., 2016). When activated, mast cells undergo degranulation with biological actions in various inflammatory settings (Galli et al., 2005; Wernersson and Pejler, 2014), and large amounts of chymase cleave many peptides and proteins, including Ang I, pro-matrix metalloproteinase-9, pro-IL-1β, pro-IL-18, and IL-6 (Urata et al., 1990; Mizutani et al., 1991; Tchougounova et al., 2005; Fu et al., 2017). Thus, detrimental impact of mast cells has been reported in settings like atherosclerosis, cardiac dysfunction, and abdominal aortic aneurysms (Sun et al., 2007; Wei et al., 2010; Kovanen and Bot, 2017; Tomimori et al., 2019). Chymase has been shown to predominate over ACE activity in human heart, accounting for extremely high total Ang II formation in the human heart, as compared to other species (Balcells et al., 1997). Local formation of Ang II is increased in aged hearts, and chymase is primarily responsible for this increase (Froogh et al., 2017). Accordingly, chronic ACE inhibition did not repress Ang II levels in the cardiac interstitial fluid while combined chymase and ACE inhibition improved cardiac function, decreased adverse cardiac remodeling, and improved survival after myocardial infarction (Wei et al., 2010). In line with these findings, knockout of ACE resulted in revoked formation of Ang II in the circulation, while the formation of Ang II in the heart was not suppressed (Wei et al., 2002). Others have shown protective effects of chymase inhibition in various cardiac conditions like myocardial ischemia-reperfusion injury, inflammation after acute myocardial ischemia/reperfusion, cardiac fibrosis, cardiac function after left ventricular repair, in cardiac function and survival after myocardial infarction, and in preventing cardiac fibrosis and improving diastolic dysfunction in the progression of heart failure (Jin et al., 2003; Matsumoto et al., 2003; Kanemitsu et al., 2008; Oyamada et al., 2011; Maeda et al., 2012; Hooshdaran et al., 2017). Although, chymase inhibitors have been shown to function in animal models, these drugs have failed in early clinical testing, possibly due to differences in chymase genes and hydrolytic activity of chymase isoforms influencing the action of mast cells proteases (Ahmad and Ferrario, 2018). Important to note is also that the findings of chymase have largely been obtained in homogenized tissues, which means that the physiological relevance in humans can be difficult to assess. The challenge is to clarify the mechanisms of intra- and extracellular chymase activation. At present, several newly developed chymase inhibitors are evaluated in pre-clinical studies (Dell'Italia et al., 2018).

### The ACE2 – Ang-(1–7) – Mas Axis

Ang-(1–7) is derived from Ang II through the influence of angiotensin ACE2, and exerts its effect *via* the G-protein-coupled Mas receptor (Santos et al., 2003). The axis of ACE2 – Ang-(1–7) – Mas opposes effects of the ACE – Ang II – Ang II type 1 receptor (AT1R) axis and has been proposed to have a protective effect (Ferreira et al., 2012), and increased Ang-(1–7) has been associated with a favourable phenotype, with attenuated inflammation in atherosclerotic plaques (Fraga-Silva et al., 2014). Ang-(1–7) induces vasodilatation via activation of NO, and decreases fibrosis, thereby enhancing the effects of ACE inhibitors on Ang II (Bader, 2013). Ang-(1–7) may mediate anti-inflammatory and anti-thrombotic effects *via* activation of NO and inhibition of ROS, derived from nicotinamide-adenine dinucleotide phosphate oxidase (Nox) (Sampaio et al., 2007; Gwathmey et al., 2010). Although animal experiments suggest the ACE2 – Ang-(1–7) – Mas axis to be an important counterregulatory arm within the RAAS (Santos et al., 2018), there are only few and conflicting data available in man. Gaidarov et al. found no evidence that Ang (1–7) interacts with or initiates signaling through individual expressed recombinant MAS1, but can potently antagonize AT1R signaling (Gaidarov et al., 2018). In addition, Ang-(1–7) has been shown to cause vasodilation in the forearm circulation in normotensive and hypertensive subjects (Sasaki et al., 2001) and to potentiate the vasodilatory effect of bradykinin, possibly through NO release, in forearm resistance vessels (Ueda et al., 2001); however others observed no vasodilatory effects by Ang-(1–7) in the human forearm (Wilsdorf et al., 2001).

### The Angiotensin II Type 1 and 2 Receptors

Ang II primarily exerts its influence through the AT1R and AT2R. The expression of the AT2R is limited in adults, but is increased after vascular injury and after myocardial or renal tissue injury (Matavelli and Siragy, 2015). The main effects of Ang II by activation of AT1R include vasoconstriction by VSMC stimulation, sodium retention in the kidneys, aldosterone release, cell proliferation with vascular and cardiac hypertrophy and fibrosis, inflammation, oxidative stress, and increased activity of the inflammasome NLRP3, promoting inflammation (Marcus et al., 2013; Whaley-Connell et al., 2013; Wen et al., 2016). In that aspect, AT1R antagonists may reduce inflammation and myocardial fibrosis after acute myocardial infarction by signaling pathways of NF-κB and TGF-β1 (Song et al., 2014), and supress increases in myocardial mRNA expression of proinflammatory cytokines (IL-6, IL-1β), MCP-1 and matrix metalloproteinases-2 and -9 in chronic heart failure (Sukumaran et al., 2010). Also, the AT1R antagonist telmisartan reduced rat cardiac myofibroblast secretion of matrix metalloproteinases-9 during stimulation with Ang II and IL-1β, while an AT2R antagonist, had no effect (Okada et al., 2010). Clinical studies have confirmed that ACE inhibitors and AT1R blockade can modify cardiac remodeling (Malmqvist et al., 2001; Dell'Italia, 2011; Ferrario, 2016), and the ACE inhibitor perindopril reduced the levels of IL-1β in patients with stable coronary disease and in essential hypertension (Madej et al., 2009; Krysiak and Okopień, 2012), implicating that ACE inhibitors may attenuate inflammasome activity.

AT2R-mediated effects generally oppose those effects mediated by AT1R, and include vasodilatation and anti-inflammatory effects in VSMCs, but also antiproliferative effects in vessels and the myocardium (Qi et al., 2012). The selective AT2R agonist compound 21 reduces proliferation, inflammation, remodeling, and fibrosis (Wang et al., 2017; Sumners et al., 2019). The anti-inflammatory effects by compound 21 reduces expression of cytokines such as IL-1β and TGF-1β, suggesting a reduced effect of the inflammasome NLRP3 to prevent abdominal aortic aneurysm progression in rat (Lange et al., 2018). Also, experimental myocardial infarction in Wistar rats and levels of cardiac IL-1β, IL-6, and IL-2 were associated with antiapoptotic and anti-inflammatory mechanisms by compound 21 (Kaschina et al., 2008), and compound 21 prevented vascular inflammation *in vitro* and *in vivo* (Sampson et al., 2016).

Aldosterone is produced in the adrenal cortex and acts on sodium reabsorption in the kidney. Ang II stimulates aldosterone synthesis by activation of the adrenal AT1R (Rautureau et al., 2011); further common stimuli are high plasma levels of potassium and adrenocorticotropic hormone. Aldosterone is implicated in vascular inflammation, oxidative stress, fibrosis, remodeling and endothelial dysfunction, particularly in the presence of salt (Park and Schiffrin, 2002). Conversely, mineralocorticoid receptor blockers reduce the effects on vascular remodeling (Virdis et al., 2002). In VSMCs and in endothelial cells, aldosterone exerts its effects *via* mitogen-activated protein kinase (also known as extracellular signal regulated kinase) and cellular Src kinase (a non-receptor tyrosine kinase), and participates in epidermal growth factor receptor transactivation (Mazak et al., 2004; Nakano et al., 2005). There is cross-talk between aldosterone and Ang II in VSMCs (Min et al., 2007). Aldosterone induced increase in oxidative stress in VSMCs may have a negative impact on endothelial function through a reduction in NO bioavailability (Nakano et al., 2005). Vascular inflammation in endothelial cells is promoted by aldosterone induced expression of ICAM-1 and adhesion of leukocytes in a mineralocorticoid receptor dependent manner (Caprio et al., 2008).

## Angiotensin II and Oxidative Stress

### Angiotensin II Generates a Proinflammatory Phenotype

A cascade of intracellular signaling responses is initiated when Ang II binds to the AT1R to cause oxidative stress and reduce NO activity. Ang II is a powerful activator of vascular Nox, which induces the production of ROS (H₂O₂) from endothelial cells and VSMCs (Santillo et al., 2015) to act as a second messenger to stimulate multiple signaling molecules (Touyz et al., 2003; Nguyen Dinh Cat et al., 2013). Several isoforms of Nox have been identified (Lambeth, 2004). Vascular endothelial cells express Nox 1, 2, 4, and 5 (Damiano et al., 2013), while Nox 2 is present in VSMCs of resistance arteries (Lassègue and Clempus, 2003), and Nox 1 and 4 are the main isoforms in VSMCs of large arteries (Hilenski et al., 2004). The isoforms Nox 2 and 4 are widely expressed in cardiomyocytes (Byrne et al., 2003; Murray et al., 2013). Nox 1 may play a major role in Ang II mediated impairment of kidney function by increasing oxidative stress (Zimnol et al., 2020). Reactive oxygen species also increase intracellular calcium and activate NF-ҡB and activating protein-1. These molecules participate in migration and cell-growth, and in the expression of inflammatory genes and extracellular matrix modulation. Ang II also activates ras homolog gene family, member A, which is important in vascular contraction and growth (Loirand and Pacaud, 2010).

**FIGURE 4:**
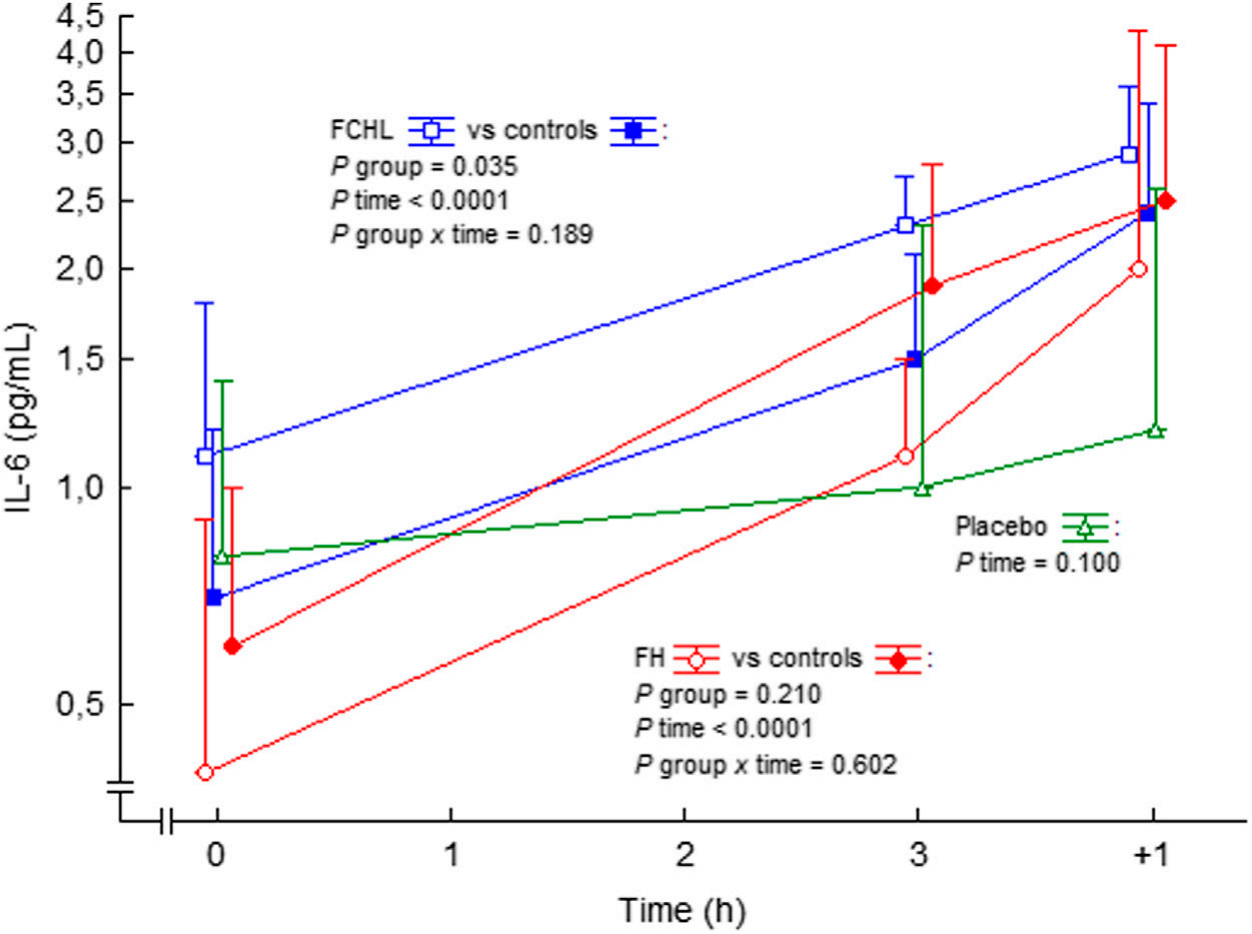
IL-6 in plasma before, during and after intravenous 3 h steady state angiotensin II infusion in man. Data are presented as median values and interquartile ranges. Statistical evaluation was made by repeated measures ANOVA. PAI-1, plasminogen activator inhibitor-1; FCHL, familial combined hyperlipidemia, and FH, familial hypercholesterolemia. FCHL, blue line and unfilled squares (*n* = 16); FCHL-control, blue line and filled squares (*n* = 16); FH, red line and unfilled circles (*n* = 16), and FH-control, red line and filled circles (*n* = 16). The effect of physiological saline infusion in placebo experiments, green line and unfilled triangles (*n* = 8), is also shown. Data from (Ekholm et al., 2009; Ekholm et al., 2015).

In man, systemic Ang II infusion increases circulating IL-6 in healthy subjects and patients with familial combined hyperlipidemia and familial hypercholesterolemia (Ekholm et al., 2009; Ekholm et al., 2015) (Figure 4). IL-6 may have reached even higher levels at local areas in vessels, which may represent a local increase of trans-signalling, and a proinflammatory effect (Rose-John, 2012). Thus, circulating Ang II may have proinflammatory effects also in man.

## Angiotensin II and Tissue Factor

### Angiotensin II Up-Regulates Tissue Factor

There is evidence that Ang II induces synthesis of TF, and that the promotor gene of TF is under control of the key redox-sensitive transcription factor NF-kB (Mackman, 1997; Felmeden et al., 2003; He et al., 2006; Li et al., 2009). Ang II binding to AT1R causes activation of NF-kB and upregulates TF synthesis in human monocytes (He et al., 2006), rat aortic endothelial cells (Nishimura et al., 1997), VSMCs (Taubman et al., 1993), and human glomerular endothelial cells (Felmeden et al., 2003). In addition, platelet activation by circulating Ang II may have an important contributing role in the generation of thrombin (Larsson et al., 2000). This is supported by findings *in vitro* and *in vivo* that Ang II stimulates formation of procoagulant microparticles from endothelial cells and mononuclear cells (Burger et al., 2011; Cordazzo et al., 2013).

### The Effects of Blocking the RAAS on Tissue Factor

Some clinical effects of ACE inhibitor therapy may possibly be caused by interrupting Nox-derived ROS, and studies have shown antioxidant effects of AT1R blockers. Thus, direct inhibition of Nox and others ROS modulators has emerged as an attractive strategy to improve endothelial dysfunction and vascular damage in hypertensive patients (Cifuentes-Pagano et al., 2014). Blocking the RAAS with ACE inhibitors (idrapril, fosinopril, or captopril) downregulated TF synthesis in monocytes (Soejima et al., 1999; Napoleone et al., 2000), while the ACE inhibitor enalapril and the AT1R blocker losartan reduced TF activity and NF-kB translocation into the nuclei in human cytokine-activated endothelial cells (Nestoridi et al., 2008), and losartan inhibited TF activity (Napoleone et al., 2000), TF mRNA synthesis in rat aortic endothelial cells (Nishimura et al., 1997) and in human glomerular endothelial cells (Nestoridi et al., 2008). In man, AT1R blockade (losartan, irbesartan, candesartan) all reduced blood pressure and TF activity, as compared to placebo, indicating inhibition of TF synthesis (Koh et al., 2004). Moreover, the direct renin inhibiter aliskiren reduced TF synthesis and function in a comparable manner to the ACE inhibitor zofenopril and the AT1R blocker olmesartan in cytokine stimulated human umbilical vein endothelial cells (Del Fiorentino et al., 2010). These results reinforce other results (Nussberger et al., 2008), and suggest that RAAS activation, *via* cross-talk between an inflamed endothelium and coagulation, contributes to a procoagulant phenotype (Levi et al., 2012).

### The Influence of Platelet Activation

Platelet activation in hypertension is important as it induces neurohumoral (sympatho-adrenal and RAAS) overactivity, endothelial dysfunction and dysfunctional NO biosynthesis, and platelet degranulation secondary to increased shear stress (Gkaliagkousi et al., 2010). Platelet activation, TF and hypertension may be linked by P-selectin. The P-selectin molecule, an integral membrane protein that is stored in platelet α-granules (Blair and Flaumenhaft, 2009) or in endothelial cells Weibel-Palade bodies, is not expressed at the cell surface in absence of inflammatory stimuli. However, during stimuli P-selectin is translocated to the plasma membrane, where it functions as an adhesion molecule, which mediates the interaction of platelets and endothelial cells with circulating leukocytes (Blair and Flaumenhaft, 2009). P-selectin also has a role as a signaling molecule, capable of inducing activation of NF-ҡB and to upregulate the expression of TF (Polgar et al., 2005). Thus, platelet activation in hypertension may *per se*, lead to increased P-selectin and, in turn, to increased TF activation.

### The Effect of Blocking the RAAS on Platelet Activation

Both ACE inhibitors and AT1R blockers have impact on the RAAS. However, these drugs have important differences between them, as ACE inhibitors reduce Ang II formation, while AT1R blockers increase Ang II (Schwieler et al., 2013). However, little is known about the impact of these drugs on platelet activity. A review by Blann et al. concluded that blocking the actions of Ang II by ACE inhibitors or AT1R blockers should theoretically have direct antiplatelet effects, but the authors concluded that there was little agreement on this effect in the clinical setting, whichever drug was used (Blann et al., 2003). Thus, ACE inhibitors increased platelet reactivity by enhanced PAR-1 expression on platelets (ref). In addition. endogenous thrombin potential was reduced by treatment with ACE inhibitors but not by ATR1 blockers (Helten et al., 2020). Similar results were obtained with ACE inhibitors and ATR1 blockers in patients with acute coronary syndromes treated with dual antiplatelet therapy (Tscharre et al., 2020).

## The RAAS and Thrombin Generation in Man

### The Effects on Thrombin Generation by RAAS Blockade in Humans

**FIGURE 5:**
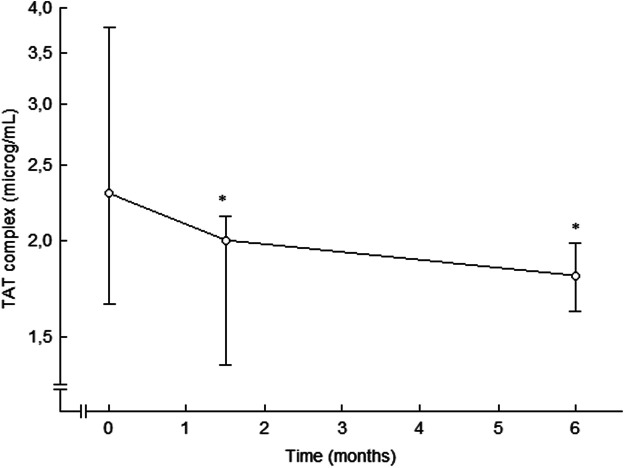
Plasma levels of TAT complex in patients with essential hypertension during placebo (0 months) and after 6 weeks and 6 months of ramipril therapy. Data are presented as median and interquartile ranges, *n* = 15. Statistical evaluation was made by non-parametric test, Wilcoxon sign test. Significant differences are given as; *p < 0.05, compared to placebo. TAT, thrombin-antithrombin. From (Ekholm et al., 2002).

Treatment with the ACE inhibitor ramipril decreased thrombin generation, as compared to placebo, in essential hypertension (Ekholm et al., 2002), (Figure 5). Thrombin-antithrombin (TAT) complex levels, a marker of thrombin generation *in vivo*, were attenuated after 6 weeks of treatment, and the effects were retained after 6 months of therapy. This attenuated thrombin generation by ramipril treatment could be of importance for the reduction in thromboembolic events seen during treatment with ramipril in patients at high cardiovascular risk (Yusuf et al., 2000). However, the antithrombotic effects of ramipril might have been due to the reduction in blood pressure *per se*.

**FIGURE 6:**
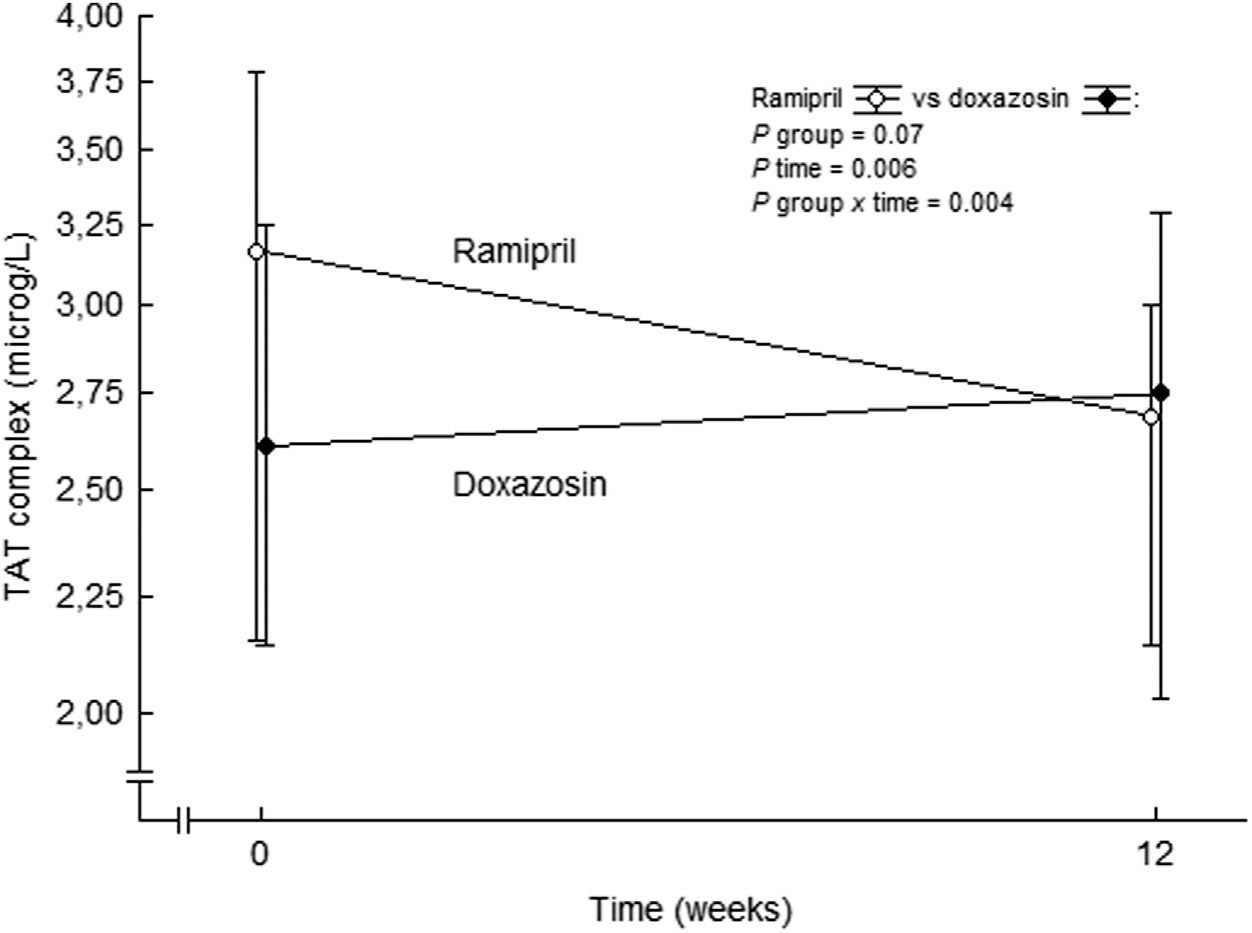
TAT complex levels at baseline and after 3 months of double-blind treatment with ramipril or doxazosin in patients with essential hypertension. Data are presented as mean values ± SD, *n* = 28 for the ramipril group and *n* = 22 for the doxazosin group. Statistical evaluation was made by multivariate analysis of variance. TAT, thrombin-antithrombin. From (Ekholm et al., 2018).

This was further assessed comparing ramipril treatment with the alpha 1-adrenoceptor blocker doxazosin. Ramipril reduced thrombin generation beyond the effects on blood pressure reduction alone (Ekholm et al., 2018) (Figure 6). TAT complex was unaffected in the doxazosin group, the reductions in blood pressure between the groups were comparable, and there was no relation between changes in TAT complex and changes in blood pressure by treatment. Thus, the antithrombin effects of ramipril are not likely to be related to reduction in blood pressure. Our findings of reduced plasma levels of TAT complex following treatment with ramipril is in line with a decreased expression and activity of TF by ACE inhibition, resulting in an attenuated thrombin generation in plasma. This confirmes our previous findings (Ekholm et al., 2002) and offers one possible mechanism by which ACE inhibitors may reduce atherothrombotic complications in high-risk cardiovascular patients (Yusuf et al., 2000; Sleight et al., 2001). Possibly the reduced levels of TAT complex reflect an attenuated thrombin generation which, at least partly, is due to a reduced expression of TF on microparticles originating from endothelial cells and/or blood cells.

### The Effects on Thrombin Generation During Angiotensin II Infusion in Humans

**FIGURE 7:**
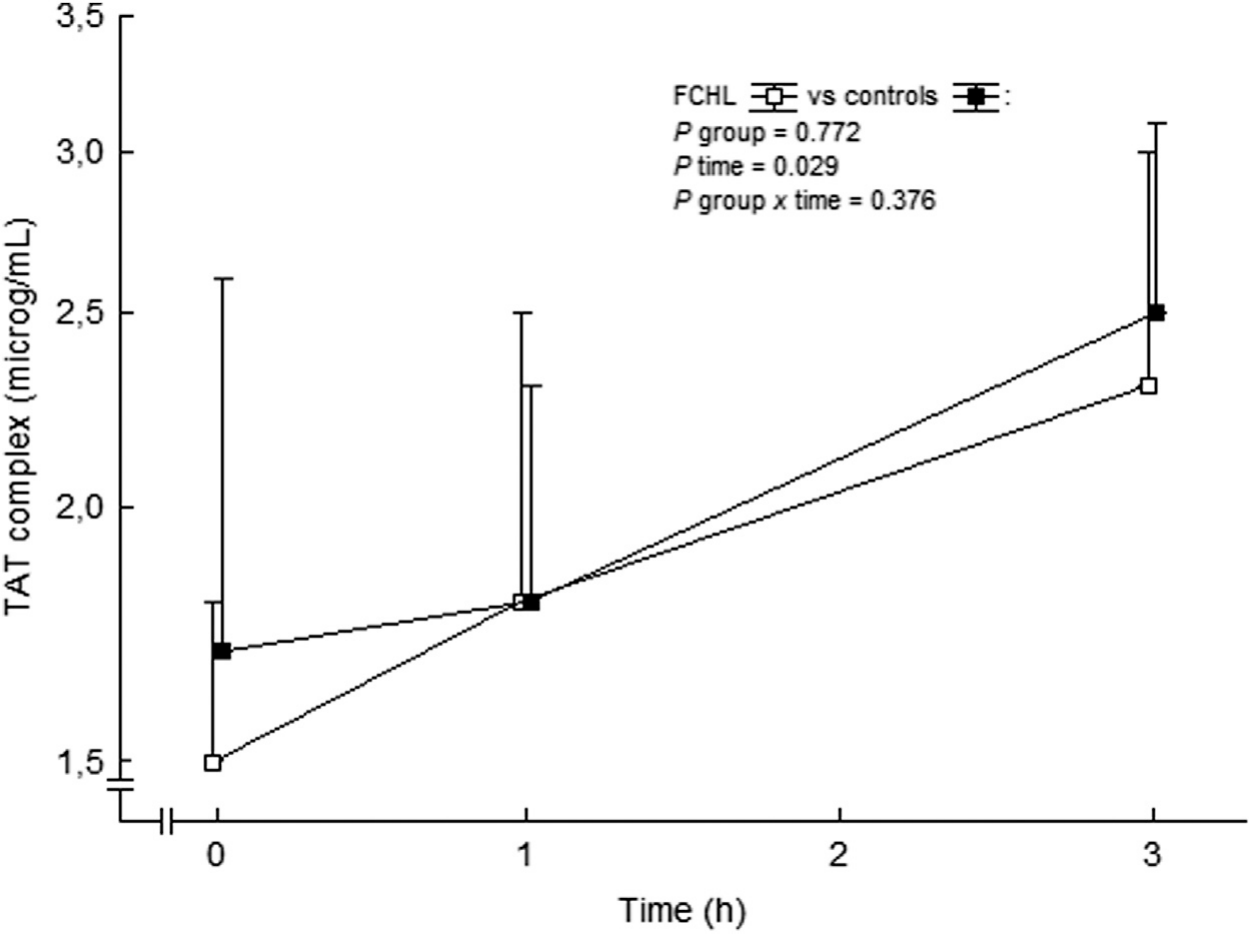
TAT complex in plasma before and during the *ongoing* angiotensin II infusion. Data are presented as median values and interquartile ranges. Statistical evaluation was made by repeated measures ANOVA. TAT, thrombin-antithrombin and FCHL, familial combined hyperlipidemia. FCHL □ (*n* = 16) and FCHL-control ■ (*n* = 16). From (Ekholm et al., 2009).

We have reported unchanged F1+2 and TAT complex values during systemic intravenous 3 h infusion of Ang II in subjects with familial combined hyperlipidemia, familial hypercholesterolemia and control subjects (Ekholm et al., 2009; Ekholm et al., 2015). This is in contrast to a previously reported increase in TAT complex and a tendency to increased F1+2 during short-time Ang II infusion in healthy males (Larsson et al., 2000). However, F1+2 exhibits a diurnal decrease during morning hours (Kapiotis et al., 1997; Ekholm et al., 2016) and these possible effects of diurnal variations of these markers were initially not taken into account by us (Ekholm et al., 2009; Ekholm et al., 2015). The absence of the expected diurnal decreases in F1+2 concentrations during morning hours, during stimulation by Ang II, may be considered as a relative increase in thrombin generation, similar to findings in healthy subjects (Larsson et al., 2000) and in familial combined hyperlipidemia patients (Ekholm et al., 2009). Indeed, in an analysis *post hoc* TAT complex actually increased in a similar way in controls and in familial combined hyperlipidemia during the *ongoing* Ang II infusion (Figure 7). Others have shown a circadian variability of TAT complex, with the highest levels at 8 am, and the lowest at 8 pm (Budkowska et al., 2019) with no variation during daytime (Jafri et al., 1992). Taken together, circulating Ang II seems to *increase* thrombin generation.

Inflammation can prime the coagulation system, and a proposed mechanism is TF expression by IL-6 (van der Poll et al., 1994). IL-6 increased during systemic Ang II infusion. Possibly Ang II may stimulate activation of TF, at least in part due to increased concentrations of IL-6. Platelet activation by circulating Ang II (Larsson et al., 2000) may have an important contributing role in the generation of thrombin.

## Fibrinolysis and the RAAS

### The Modulation of the Fibrinolytic System

The fibrinolytic system aims to dissolve and remove clots within the circulation. The zymogen plasminogen is released from the liver and is accumulated in fibrin-rich clots. Upon activation plasminogen is cleaved to plasmin by a variety of enzymes. The main regulator of fibrinolysis is tPA released from endothelial cells. The major inhibitor of tPA and urokinase-P is PAI-1. When PAI-1 binds to plasminogen activators, inactivation of these activators take place, and consequently the activity of the fibrinolysis is supressed.

The ratio between tPA and PAI-1 modulates the fibrinolytic activity (Vaughan, 2002). The immediate response during inflammatory stimuli is a transient increase in the secretion of tPA from Weibel-Palade bodies in endothelial cells (Huber et al., 2002). This increase in fibrinolytic activation is followed by a delayed suppression of tPA production and a sustained increase in PAI-1, resulting in a suppression of fibrinolytic activity. The major regulators of PAI-1 activity at inflammatory sites seem to be cytokines, such as IL-6, IL-1β, and TNF-α (van der Poll et al., 2001; Aso, 2007). CRP has also been shown to stimulate the expression of PAI-1 (Devaraj et al., 2003). Alpha granules in platelets also contain PAI-1 that can be released upon activation, which increases PAI-1 and thereby contributes to suppression of fibrinolysis in inflammatory states.

PAI-1 appears in human blood in three different forms, active, latent (representing an inactive form) and complexed to tPA or urokinase-PA. Concentration of PAI-1 exceeds tPA by a 4:1 ratio and PAI-1 binding to tPA or urokinase-PA occurs in a ratio of 1:1, thereby effectively limiting fibrinolysis. When PAI-1 levels are increased the tPA half-life is considerably shortened, and a negative correlation is present between PAI-1 and plasmin-antiplasmin (PAP) complexes. The tPA/PAI-1 complex is stable, and eliminated from the circulation by the liver. TNF-α is important in the induction of the fibrinolytic responses, while IL-6 have been shown to be most relevant for coagulation activation (Levi et al., 1997), but also enzymes like kallikrein, FXIa and FXIIa may convert plasminogen into plasmin. Plasmin acts by degrading fibrin into d-dimers. The main inhibitor of fibrinolysis and tPA is PAI-1, while *α*2-antiplasmin and *α*2-macroglobulin inhibits plasmin. High levels of PAI-1 and tPA have been shown to predict development of a first cardiovascular event (Thögersen et al., 1998). PAI-1 has a diurnal variation with its highest concentrations at 8 am and the lowest at 2 pm (Budkowska et al., 2019).

### The Influence of the RAAS on the Fibrinolytic System

The RAAS may influence the fibrinolytic system since Ang II stimulates the production of PAI-1 by endothelial cells and VSMCs, and bradykinin (which is degraded by the ACE) stimulates the production of tPA (Brown and Vaughan, 2000). Ang II can increase protein activities of TF and PAI-1 (Nishimura et al., 1997). Ang II has also been observed to increase PAI-1 and tPA activator messenger RNA (van Leeuwen et al., 1994). Additional experiments have demonstrated that Ang IV increased PAI-1 expression, and the response exhibited a fast time and dose dependence (Kerins et al., 1995). Blocking the RAAS by ACE inhibition after myocardial infarction has been shown to improve the fibrinolytic balance (Boman et al., 2002). Ang II infusion increases the expression of messenger RNA PAI-1 in the rat (Nakamura et al., 2000).

### The Effects on the Fibrinolytic System During Angiotensin II Infusion in Humans

**FIGURE 8:**
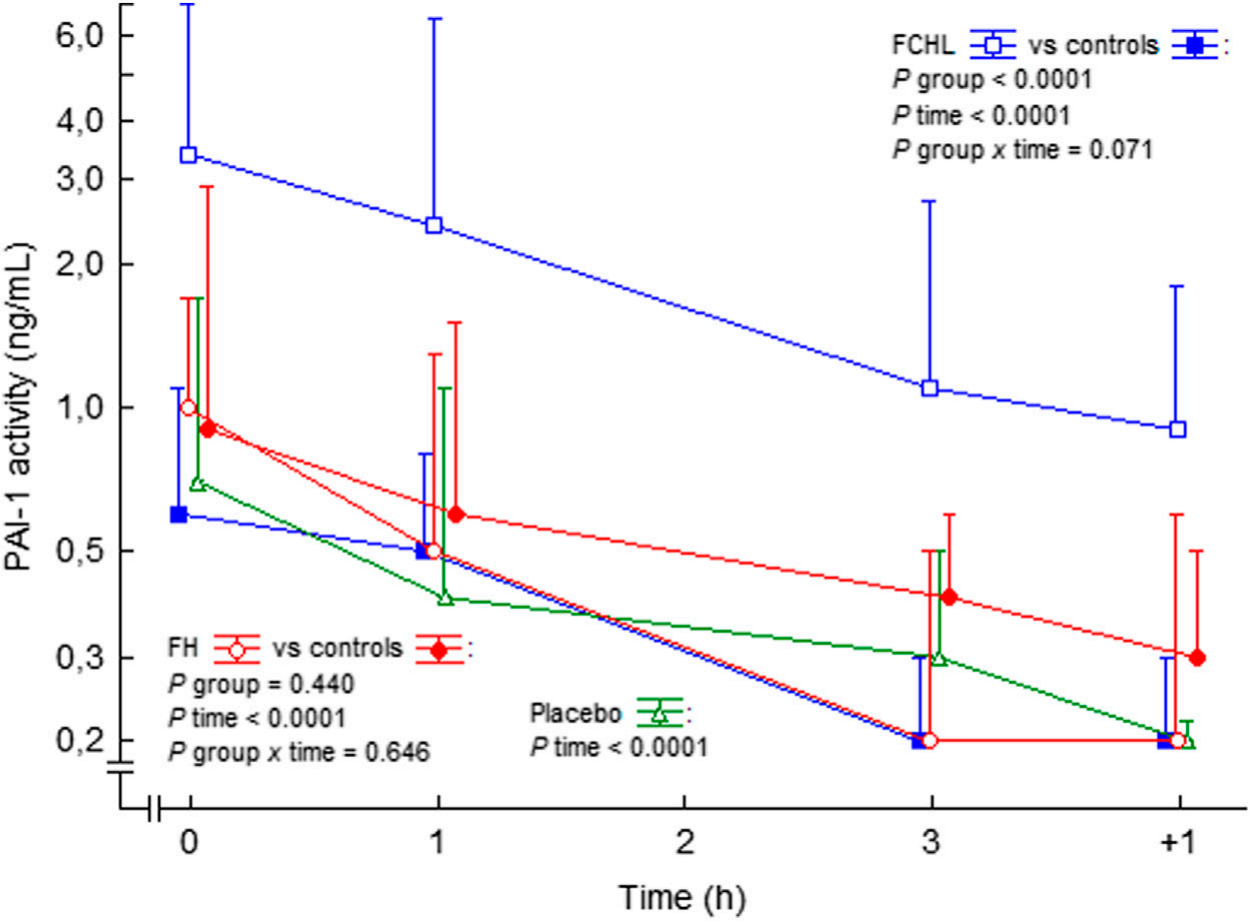
PAI-1 activity in plasma before, during and after intravenous 3 h steady state angiotensin II infusion in man. Data are presented as median values and interquartile ranges. Statistical evaluation was made by repeated measures ANOVA. PAI-1, plasminogen activator inhibitor-1; FCHL, familial combined hyperlipidemia; FH, familial hypercholesterolemia. FCHL, blue line and unfilled squares (*n* = 16); FCHL-control, blue line and filled squares (*n* = 16); FH, FH, red line and unfilled circles (*n* = 16), and FH-control, red line and filled circles (*n* = 16). The effect of physiological saline infusion in placebo experiments, green line and unfilled triangles (*n* = 8), is also shown. Data from (Ekholm et al., 2009; Ekholm et al., 2015; Ekholm et al., 2016).

In a small study with normotensive and hypertensive subjects, Ang II infusion resulted in an increase in PAI-1 antigen, whereas no changes occurred regarding tPA antigen (Ridker et al., 1993). In conflict, Ang II did not involve any changes in PAI-1 antigen or activity in healthy subjects in other studies (Larsson et al., 1999; Lottermoser et al., 2004). We observed no effects on PAI-1 activity during Ang II infusion during 3 h in control subjects, patients with familial combined hyperlipidemia, or with familial hypercholesterolemia (Ekholm et al., 2015; Ekholm et al., 2016), (Figure 8). Thus, Ang II may induce PAI-1 release in the *long-term* setting, possibly by a shift of the endothelial cells to a proinflammatory phenotype, but we could not find any *short-term* impact of Ang II on the concentrations of PAI-1. In addition, we observed that Ang II infusion also seems to induce a progressive increase in tPA activity in healthy volunteers (Ekholm et al., 2016). The response to Ang II stimulation in patients with chronic diseases such as cardiovascular disease or diabetes mellitus, which implies endothelial dysfunction, is not known.

## The Interaction Between Angiotensin II and COVID-19

### The ACE2 Receptor

The outbreak of the COVID-19 pandemic in 2019 affects individuals worldwide. The ACE2 receptor, together with the transmembrane protease serine 2, has a central role for the internalization of the virus (Liu et al., 2020a; Guzik et al., 2020). The expression of ACE2 is not limited to the respiratory system, being present also in other tissues like the gut, heart, kidney, as well as arterial and venous endothelial cells in all organs studied (Hamming et al., 2004). There is clear-cut evidence that endothelial cells are prone to acquire severe acute respiratory syndrome coronavirus 2 (SARS-CoV-2) infection (Varga et al., 2020). Preclinical studies have shown that after binding of SARS-CoV-1 and -2 to its receptor, ACE2 activates RAAS leading to downregulation of the expression of ACE2, which in turn results in excessive production of Ang II (Imai et al., 2005; Huang et al., 2014; Wang et al., 2020). Therefore, ACE2 seems to play a dual role in COVID-19. Initially, it acts as a receptor for SARS-CoV-2 entry, then, in the context of SARS-CoV-2 infection, ACE2 is downregulated, which may increase Ang II (Wang et al., 2020).

### SARS-CoV-2 Infections May Cause Inflammation and Vascular Thrombi

Recent data suggests that SARS-CoV-2 infections include vascular dysfunction, inflammation, and hypercoagulability, with involvement in multiple organs (Leisman et al., 2020). ACE2 is particularly highly expressed in pericytes, in addition to type II alveolar epithelial cells, and high expression of ACE2 in pericytes, including high levels in the heart, could lead to development of vascular dysfunction (Chen et al., 2020b; Shi et al., 2020). Vascular smooth muscle cells have both ACE2 receptor and the transmembrane protease serine 2, and lung tissue and other affected organs have revealed evidence of vascular inflammation along with vascular thrombi (Varga et al., 2020). Also, distal vasculitis with acro-ischaemic lesions have been observed at the distal aspects of toes and fingers in patients with COVID-19, and these cutaneous vasculitis lesions have been assessed as skin manifestation of SARS-CoV-2 infection (Fernandez-Nieto et al., 2020; Marzano et al., 2020; Zhang et al., 2020).

Cytokines are produced by immune cells like macrophages, dendritic cells, natural killer cells and the adaptive T and B lymphocytes. During inflammation activated macrophages will release cytokines, including IL-1β and IL-6, which stimulate the expression of adhesion molecules leading to endothelial activation and infiltration och inflammatory cells (Guzik et al., 2020). Activated endothelial cells release cytokines that may contribute to the development of microcirculatory lesions (Nencioni et al., 2009), and the dysfunctional endothelium will eventually become pro-adhesive and pro-coagulant (Boisramé-Helms et al., 2013). When SARS-CoV-2 is present in endothelial smooth muscle cells, this process is further enhanced.

### COVID-19 Infection May Cause Detrimental RAAS Activation

Patients with COVID-19 are exposed to stress, which results in RAAS activation and subsequent increased Ang II levels, which are positively associated to viral load and lung injury (Huang et al., 2014; Liu et al., 2020b). Thus, the levels of Ang II could cause an inflammatory onset with accompanying increased oxidative stress. In some patients COVID-19 is accompanied by an excessive inflammatory reaction with the release of a large amount of pro-inflammatory cytokines in an event known as a cytokine storm. Studies have suggested that the cytokine storm correlated directly with lung injury, multi-organ failure, and unfavourable prognosis of severe COVID-19 (Chen et al., 2020a; Gao et al., 2020; Ruan et al., 2020). The cytokine IL-6 has a central role in this process, and increased markers of this cytokines predict poor outcome in patients with severe COVID-19 (Cao, 2020).

During severe COVID-19 infection patients suffer from thrombotic complications to a large extent, which may partly be explained by cross-talk between inflammation and coagulation (Verhamme and Hoylaerts, 2009). When generation of thrombin occurs, this prothrombotic state in turn has a spectrum of complicating effects with endothelial dysfunction and activation of platelets, which further contributes to a prothrombotic and proinflammatory state. Localized macrophages can release pro-coagulant factors such as plasminogen activators, and with the retreat of ACE2 and activation of Ang II, the production of downstream of PAI-I is stimulated (Nishimura et al., 1997). COVID-19 patients with acute respiratory failure exhibits hypercoagulability due to hyperfibrinogenemia (Spiezia et al., 2020). This combination further accelerates vascular inflammation, and enhances a prothrombotic state, which may predispose patient to microinfarcts within multiple organs and consequently multi-organ injury and failure. A recent post mortem report of patients with COVID-19 acute respiratory distress syndrome identified severe vascular injury, including alveolar microthrombi that were nine times more prevalent than found in post mortem studies of patients with influenza dito (Ackermann et al., 2020).

### Blocking the RAAS or Not During COVID-19 Infection?

Drugs that block the RAAS may play a role in abrogating the inflammatory response, vasoconstriction and thrombotic complications that causes clinical deterioration in patients with COVID-19. We have shown that infusion of Ang II rapidly increases the levels of the cytokine IL-6 in humans (Ekholm et al., 2009; Ekholm et al., 2015), and stimulates thrombin generation. Conversely, ramipril decreased thrombin generation in hypertensive patients beyond the effects on blood pressure reduction alone (Ekholm et al., 2002; Ekholm et al., 2018). In this context, our findings would speak in favor of supressing the RAAS during COVID-19. In contrast, experimental studies have demonstrated that inhibition of the RAAS may result in a compensatory increase in tissue levels of ACE2 (Ferrario et al., 2005). Compensatory effects on ACE may lead to the RAAS being tipped towards the detrimental ACE – Ang II – AT1R axis and away from the protective ACE2 – Ang-(1–7) – Mas axis, leading to suggestions that these drugs may be detrimental in patients exposed to SARS-CoV-2 (Danser et al., 2020). However, there is no clear evidence that ACE inhibitors or AT1 receptor blockers lead to upregulation of ACE2 in human tissues (Danser et al., 2020).

A retrospective cohort study included 18.472 patients tested for COVID-19 found no association between ACE inhibitors or AT1R blockade use and a positive COVID-19 test (Mehta et al., 2020). A cohort study including 8.3 million people concluded that ACE inhibitors and AT1R blockers were associated with reduced risk of COVID-19 disease (Hippisley-Cox et al., 2020) and a meta-analysis that enrolled ten studies and 9.890 hypertensive patients strongly supported the recommendation to continue ACE inhibitors or AT1R blockade for all patients (Flacco et al., 2020). In a meta-analysis that included 25 observational studies neither ACE inhibitors nor AT1R blockers were associated with increased odds ratio for SARS-CoV-2 infection, admission to hospital, severe critical illness, admission to intensive care unit, or SARS-CoV-2 related death. In addition, the authors concluded that ACE inhibitors might be marginally protective regarding SARS-CoV-2 related death compared with AT1R blockade (Patoulias et al., 2020). More recently, a systematic review and meta-analysis of 31 cohort studies with outcome data for 87,951 patients, of whom 27% were on ACE inhibitor or AT1R blocker therapy, and three population based case control studies found no association between the use of RAAS blocking drugs and mortality, severe disease and no differential effect between ACE inhibitor or AT1R blocker therapy and outcome (Bavishi et al., 2021) However, current knowledge on this issue may be considered preliminary until confirmed in properly designed prospective randomized controlled studies. Such studies are ongoing.

## Conclusion

The cause of the increased thrombin generation in hypertension and cardiovascular disease remains unclear. However, inflammatory stimuli can prime the coagulation system. Since inflammation participates in vascular remodeling and atherosclerosis, and Ang II has an important role for inflammation in the vessels, Ang II may contribute to vascular dysfunction in hypertension. One possible mechanism could be that Ang II may stimulate activation of TF. Platelet activation by circulating Ang II may also contribute to the generation of thrombin. Antihypertensive treatment with ACE inhibitor may reduce thrombin generation beyond the effects on blood pressure reduction alone. Thus, drugs blocking the RAAS may reduce atherothrombotic complications beyond their effects to reduce blood pressure. In addition, available preliminary experimental and clinical evidence suggests that blocking the RAAS may prevent complications in COVID-19, and blocking RAAS might even exert a potentially protective influence in the setting of COVID-19. Thus, current evidence does not favor the discontinuation of RAAS blocking drugs in patients with COVID-19 and hypertension or other cardiovascular comorbidities.
